# The Centenary of Immune Thrombocytopenia – Part 1: Revising Nomenclature and Pathogenesis

**DOI:** 10.3389/fped.2016.00102

**Published:** 2016-10-19

**Authors:** Rita Consolini, Annalisa Legitimo, Maria Costanza Caparello

**Affiliations:** ^1^Laboratory of Immunology, Department of Clinical and Experimental Medicine, Division of Pediatrics, University of Pisa, Pisa, Italy

**Keywords:** immune thrombocytopenia, pathogenesis, autoimmune disease, megakaryocytopoiesis, platelets

## Abstract

The natural history of the immune thrombocytopenia (ITP) is interesting and intriguing because it traces different steps underlying autoimmune diseases. The review points out the main steps that have accompanied the stages of its history and the consequential changes related to its terminology. ITP is an autoimmune disease resulting from platelet antibody-mediated destruction and impaired megakaryocyte and platelet production. However, research advances highlight that a complex dysregulation of the immune system is involved in the pathogenesis of this condition. The review examines the role of the multiple immune components involved in the autoimmunity process, focusing on the more recent mechanisms, which could be new promising therapeutic targets for ITP patients.

## Introduction

Immune thrombocytopenia (ITP) is a blood disorder characterized by a low platelet count and mucocutaneous bleeding. The severity of bleeding generally correlates with the severity of thrombocytopenia. Its estimated incidence is 100 cases out of a million people per year; about half of these cases occurs in previously healthy children, where it represents the most frequent blood disorder. The etiology of ITP is still unknown, and the diagnosis continued to be one of exclusion: the appropriate methodological approach will be described in the second part of the review. Since the beginning of the century, its long history brought new insights about the pathogenesis of the disorder, leading to better refining of both terminology and clinical states. Although the platelet destruction by autoantibodies is considered the main pathogenetic mechanism, a complex dysregulation of the immune system is observed in ITP patients. Autoantibodies also may fix complement to platelets, T-cell and cytokine profiles are shifted to type 1 immune response, and the monocytic phagocytic system appears to be excessively activated, resulting in a pathogenic loop (1). In addition, several negative immune regulators such as T and B regulatory cells (Tregs, Bregs) and tolerogenic dendritic cells (DCs) are dysfunctional, enable to efficiently suppress the pathogenic process (1). The recently discovered T follicular helper cells (TFHs) are reported to be involved in B cell recruitment and differentiation in the spleen of ITP patients (2). Therefore, the autoimmune attack on platelets (and megakaryocytes) is amplified by multiple mechanisms. Together with the antibody suppression of megakaryopoiesis and thrombopoiesis, also other mechanisms such as abnormal apoptosis and thrombopoietin (TPO) dysregulation are described (3–7). Further, genetic factors are thought to play a role in susceptibility to developing ITP (8, 9). The knowledge of the multiple immune defects has opened new therapeutic opportunities that we will discuss in the next second part of the review.

## The Intriguing History of ITP

The history of ITP is interesting and intriguing because it traces the different steps underlying autoimmune diseases. It starts with the identification of platelets into the blood, goes round the definition of the immuno-pathogenetic mechanisms, and ends by opening the window for new therapeutic options in the field of autoimmune disorders (Table 1). Many evolving concepts have subtended the pathophysiology of ITP. The historical perspective of ITP has been detailed in the outstanding review of Stasi and Newland (10), where the readers can found exciting suggestions for the comprehension of developing steps of ITP pathophysiology, as if it were a model of autoimmune disorder. Our review summarizes the essential points that mark the history of this disease. In 1915, Frank proposed that ITP resulted from the suppression of platelet production by megakaryocytes (MKs), due to a toxic factor produced by the spleen. The following year, based on the evidence that splenectomy normalized the platelet count in many patients with ITP, a medical student called P. Kaznelson, radically changed Frank’s hypothesis. He suggested that thrombocytopenia was due to an increased platelet destruction in the spleen, focusing on the critical role of the spleen in the pathogenesis of ITP. Furthermore, the mechanism of the spleen platelet destruction remained to be clarified, if by the direct destruction or by the release of an inhibiting factor. In 1938, Troland and Lee experienced that the injection of a substance (thrombocytopen) into the rabbits, extracted from spleen of patients with ITP, consistently produced a rapid, although transient, fall of the platelet count. Despite of this, other Authors affirmed that the disease was due to a fundamental abnormality of the spleen, which “exerted an unusual effect upon the production of platelets from the megakaryocytes in the marrow” (11). In 1951, Harrington–Hollingsworth experiments unequivocally settled the debate about the mechanism of the ITP thrombocytopenia, i.e. peripheral platelet destruction versus impaired platelet production. They demonstrated that healthy volunteers (Harrington included!), who received a blood transfusion from patient with ITP, became thrombocytopenic, concluding that ITP was characterized by reduced platelet survival due to a plasmatic factor. In the same year, Evans et al. suggested that the thrombopoietic factor was an antiplatelet antibody, identified as immunoglobulin (Ig) of G isotype (12). Its increase was subsequently demonstrated in 90% of chronic ITP patients (13). In 1982, new investigations about the antibody components of ITP, performed by Van Leeuwen et al., demonstrated that ITP patients produced autoantibodies against two platelet glycoproteins (GP), GPIIb and GPIIIa. Since that time, several laboratories have provided direct evidence for the presence of autoantibodies against GPIIb/IIIa and other platelet antigens in PTI (14–17). In 1994, the primary regulator of platelet production, called thrombopoietin, was purified and cloned (18–20); its role in the pathophysiology of ITP, underlying inappropriate thrombopoiesis in most patients, has been suggested (21, 22). In 1996, Wright et al. elucidated the cross-reactive nature of these autoantibodies, introducing in the ITP pathogenesis, the hypothesis of “molecular mimicry,” resulting in loss of tolerance and the activation of the autoimmunity mechanisms. Since the early 1980s, several studies have supported the hypothesis that thrombocytopenia might result not only from antibody platelet destruction but also from antibody against megakaryocytes (23–25). The role of autoantibodies in patients with ITP has been further defined by *in vitro* experiments, supporting the view that autoantibodies in ITP either suppressed MKs production or maturation and platelet release (26, 27). Other investigators confirmed the involvement of megakaryocytes, by showing in ITP patients, extensive megakaryocytic abnormalities suggestive of a mechanism of “non-classical apoptosis” (28). Already since 1991, Semple et al. suggested that not only the antibody response but also T cell compartment was involved with multiple abnormalities in the pathogenesis of ITP. In particular, they showed that the disorder might be the result of an abnormal T helper (Th) cell defect, that could direct autoreactive B cells to produce autoantibodies. Therefore, ITP was considered a disease due to an immune system multi-dysfunction, in which not only the T helper cells were involved, but also T regulatory cells that might play a role in the persistence of disease through the loss of tolerance (29, 30). Olsson et al., in 2003, brilliantly discovered that cytotoxic T (Tc) cells in ITP patients, deprived of platelet antibodies, could cause thrombocytopenia (31). Subsequent studies in 2007, suggested that CD8^+^ T cells lead to impaired platelet production, by suppressing autologous MK apoptosis (32). From the year 1951, that marked the beginning of the era of the immunologic character of ITP, focusing as central theme “the autoantibody-platelet destruction,” ITP pathogenesis appears to be multifactorial, resulting as a complex interplay involving multiple components of the immune system. The considerable advances in immunology over the past two decades expanded our knowledge about the immune defects underlying the ITP pathogenesis and allowed the development of “targeted therapies.”

**Table 1:** **The historical view of ITP**.

Year	Author	Main discovery
1915	Frank (33)	Toxic factor produced by the spleen
1916	Kaznelson (34)	Increased platelet destruction in the spleen
1938	Troland and Lee (35)	“Thrombocytopen” factor in the spleen
1946	Dameshek and Miller (11)	Abnormality of the spleen
1951	Harrington et al. (36)	Plasmatic factor
1951	Evans et al. (37)	Antiplatelet autoantibodies
1965	Shulman et al. (12)	Isotype of autoantibodies
1975	Dixon et al. (13)	
1978	McMillan et al. (23)	Anti-megakaryocyte autoantibodies
1982	Van Leeuwen et al. (38)	Anti-glycoprotein autoantibodies
1991	Semple et al. (39)	T cell abnormalities
1994	Bartley et al. (18)	Thrombopoietin
	Kuter et al. (19)	
	Lok et al. (20)	
1996	Wright et al. (40)	Molecular mimicry
2003	Olsson et al. (31)	Cytotoxic T cell involvement
2003	Chang et al. (26)	Anti-megakaryocyte autoantibodies
2004	McMillan et al. (27)	
2004	Houwezijl et al. (28)	Megakaryocytic abnormalities
2005–2016	Involvement of multiple components of the immune system in a complex interplay	

## New Terminology and Changes of Definition of the Different Phases of ITP

**Figure 1:**
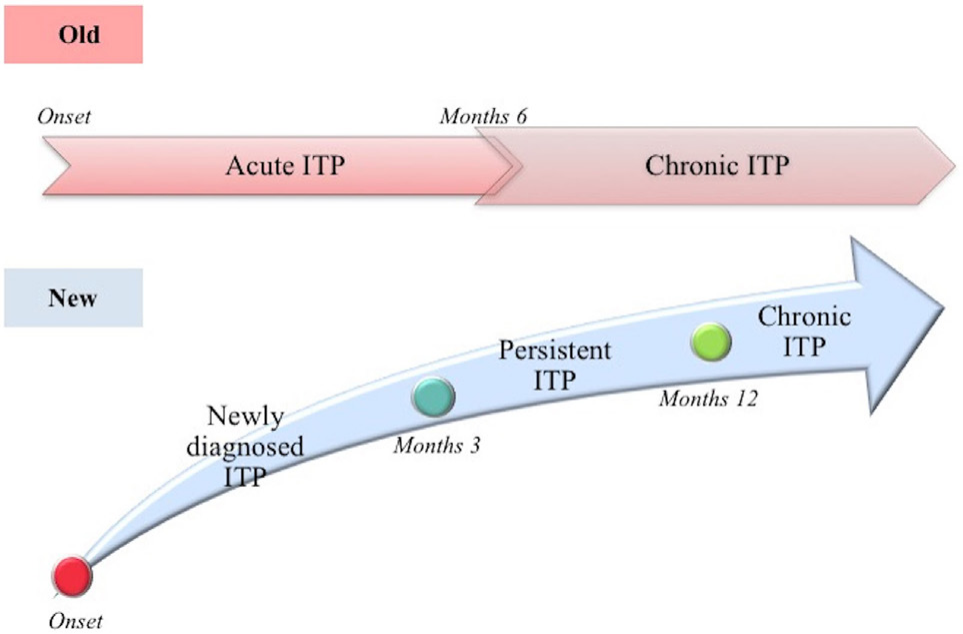
**Old and new definition of the different stages of disease (immune thrombocytopenia)**.

Based on the above described story, in 2009, the International Working Group (IWG), following the Consensus Conference in Vicenza, Italy (the Vicenza Consensus Conference, October 2007), proposed a new terminology, with the aim to underline the autoimmune pathogenesis of ITP [(41); Figure 1]. IWG decided to maintain the acronym ITP, recognizing its historical origin, but changing the meaning. The acronym ITP means “IMMUNE THROMBOCYTOPENIA”: the letter “P” today means “Primary” and replaces the outdated “Purpura,” which is considered an inappropriate term to describe the disorder, being the symptoms (like purpura) absent in most cases. Instead, the term “primary” refers to the absence of a recognized cause of the disease. Additionally, the IWG clearly distinguished between primary and secondary forms, in which thrombocytopenia is due to an underlying disease present at diagnosis (42). Unless otherwise specified, ITP refers to the primary form of immune thrombocytopenia. Along with the change of terminology concerning the disease definition, also the definition of its different phases has been changed. Traditionally, the definitions “acute ITP” and “chronic ITP” have been used to identify respectively a self-limited form of the disease (e.g., secondary to viral infection in children) and a lasting for more than 6 months form. Currently, as reliable predictive clinical and laboratory signs of disease duration have not yet been identified, the term “*newly diagnosed ITP*” is used to define all cases at diagnosis (41). In most cases (85%), the disease is self-limited, reaching a complete remission in 3–6 months from diagnosis. It is typically observed in children, both male and female with the same incidence, showing (in 50–60% of the cases) a recent history of viral disease. Twenty percent of cases do not achieve spontaneous remission or do not reach or maintain remission after treatment in the period lasting between 3 and 12 months from diagnosis; the new term “*persistent ITP*” is introduced to define these ITP patients. Furthermore, the term “*chronic ITP*” is used for patients with ITP lasting for more than 12 months. The only term that is historically maintained is “*refractory ITP*,” referred to the chronic form that is not responsive to the treatment.

## Pathogenesis

Immune thrombocytopenia is historically considered an autoimmune disease attributed to platelet destruction due to antibodies secreted by autoreactive B cells. Moreover, continued research provided evidence that an impaired platelet production is also important in the pathogenesis of ITP, leading new insights into the comprehension of the complex immunopathogenic mechanisms underlying this disease (43, 44). The triggering event of ITP is unknown, and it is still unclear whether the immune defects associated with ITP play causative roles in the disease or are secondary epiphenomena (evoked by the structural platelet glycoprotein modifications) brought on by the inflammation associated with the disorder (44). Furthermore, a complex array of the immune mechanisms involved in the ITP autoimmunity has been delineated in the recent years.

### Effects of Autoantibodies on Platelets and Megakaryocytes

Classically, the primary event of ITP is the arising of autoantibodies opsonizing platelets that leads to a markedly enhanced Fc-receptor (Fc-R)-mediated clearance of platelets by the monocytic phagocytic system (macrophages and dendritic cells) residing in the spleen (and liver) (43). Both platelet destruction and impaired platelet production are primarily mediated by IgG autoantibodies, although IgM and IgA may be involved (45, 46), mainly directed against platelet membrane glycoprotein complexes, such as GPIIb/IIIa and GPIb/IX (2, 47); other GP (Ia–IIa, IV, and V) have also been identified (46). Furthermore, it has been demonstrated that the two most frequent platelet membrane glycoprotein targets, GPIIb heterodimer and GPIb/IX complex, were also expressed on megakaryocytes during the early stages of differentiation (48). Moreover, autoantibodies were also found to bind MKs (23). In the late 1960s, animal models provided the first demonstration that autoantibodies might not only cause peripheral destruction of mature platelets but also directly affect MKs. Subsequently, in a study of pediatric patients suffering from ITP, a significant suppression of *in vitro* MK production was observed, when the plasma samples contained anti-GPIb/IX antibodies alone or in combination with anti-GPIIb/IIIa antibodies (26). By using MK cultures derived from human CD34^+^ cell, it has been recently demonstrated that antiplatelet antibodies inhibited proplatelet formation by MK and, therefore, their ability to release platelets (49). Additionally the discover of an autoantibody-antigen target, the Mpl TPO receptor (the cellular homolog of the myeloproliferative leukemia virus oncogene), allowed to demonstrate, by using plasma derived from thrombocytopenic ITP patients in MK colony-forming assays, the suppression of MK proliferation by anti-Mpl autoantibodies (50).

### The Role of Complement

There are clear indications that complement activation is involved in the disappearance of platelets from the circulation of patients with ITP (51), although its role and that one of complement receptors in the pathogenesis of ITP are poorly understood (52). The autoantibodies, specifically bound to platelet antigens, can fix complement (C3) on platelet and megakaryocyte (MK) membranes, triggering cell destruction through complement system, either by enhancing clearance or direct cell destruction. Subsequent studies have described the intrinsic ability of platelets to activate both classical (53, 54) and alternative (52, 55) pathways of complement.

### Other Mechanisms of Impaired Thrombopoiesis

**Figure 2:**
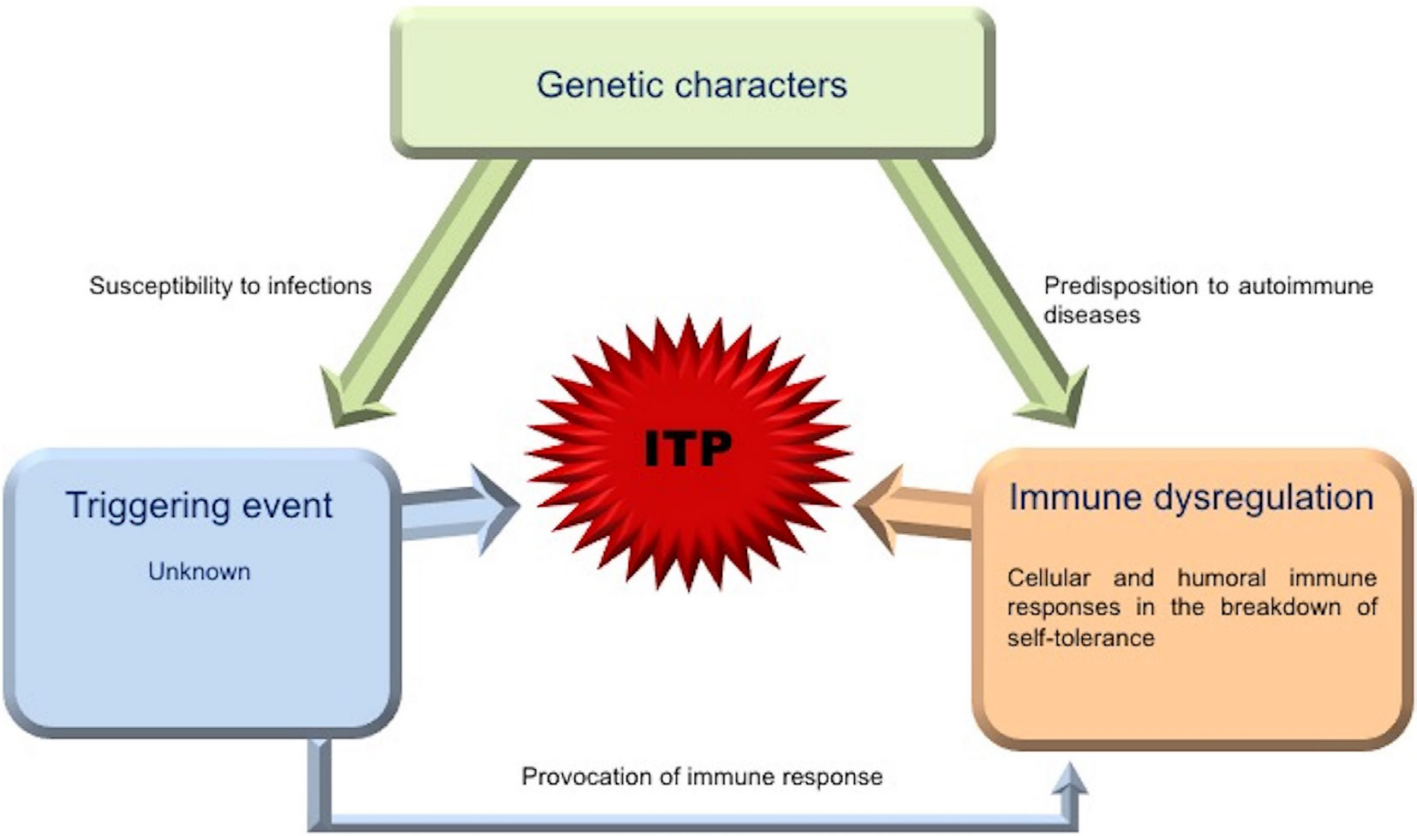
**Picture of interplay of contributing factors in ITP**. The triggering event for ITP is substantially unknown. Genetic factors may play an important role in the development of the disease, being associated with increased susceptibility to infections or predisposition to autoimmune diseases. The generalized immune dysregulation, with an imbalance between the immunoregulatory elements (cells, receptors, cytokines, and other signaling molecules), regulatory and effector T cells, drives pathogenic T and B cell effector responses against platelets and megakaryocytes.

**Figure 3:**
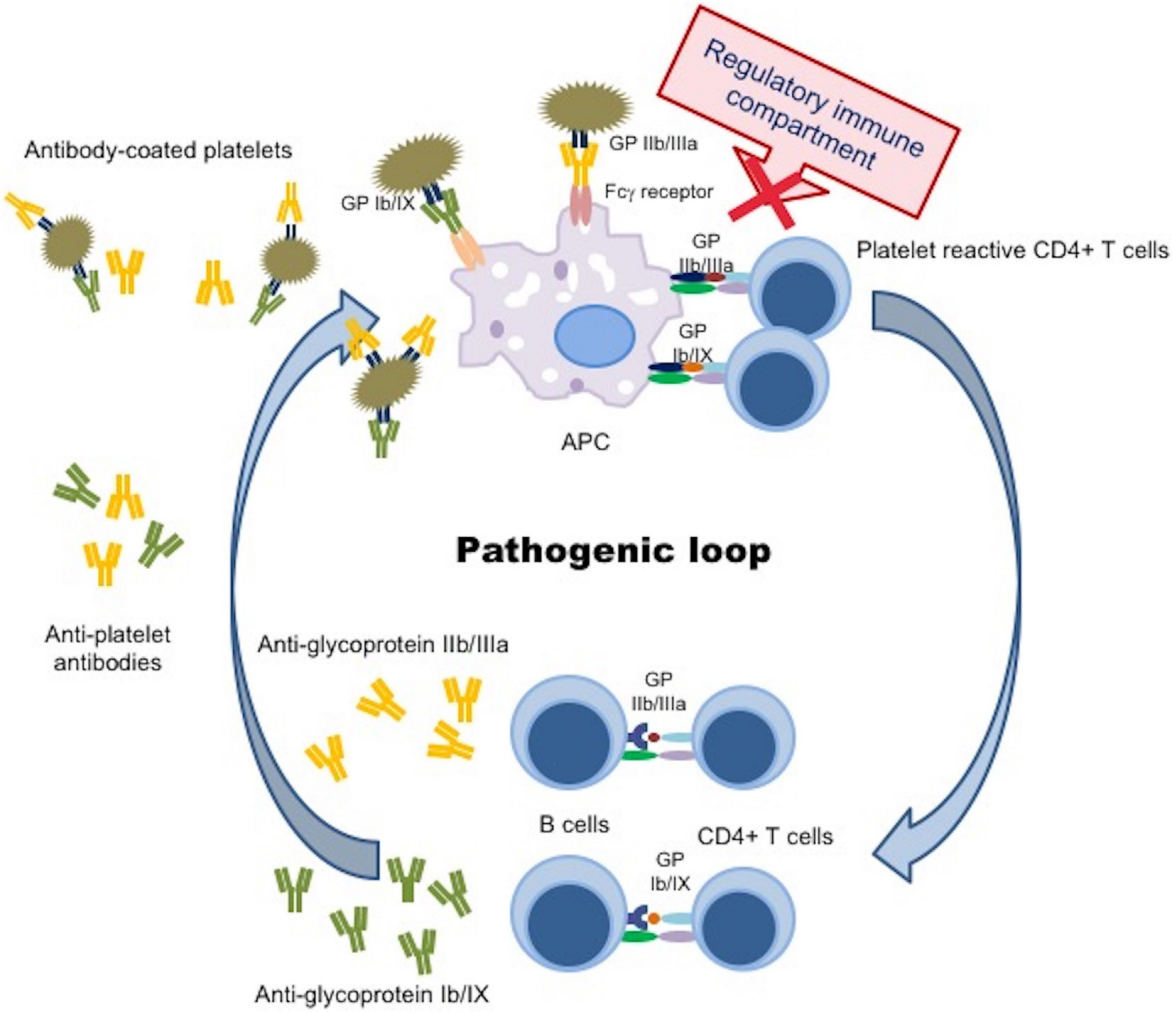
**Pathogenic loop in immune thrombocytopenia**. Schematic picture of a continuous pathogenic loop carried out by APC, autoreactive CD4^+^ T cells, and autoantibody-producing B cells that maintains antiplatelet antibody production in ITP patients. A defect in the immune regulatory compartment (DCs, Tregs, Bregs) did not suppress the interaction between APC and autoreactive CD4^+^ T cells, resulting in prevention from a trigger of the pathogenic loop. APC, antigen presenting cell; GP, glycoprotein.

Platelet kinetic studies suggested that both impaired platelet production/destruction might be determined not only by autoantibody suppression of both megakaryopoiesis and thrombopoiesis, but also by other mechanisms such as abnormal apoptosis and TPO dysregulation (3, 4). Although an increased number of mature megakaryocytes compared to normal controls are observed in ITP patients, the maturation program of these cells appears to be impaired. The histopathology and ultrastructural abnormalities of bone marrow of the majority of patients are consistent with an apoptosis-like programed cell death (28). Elevated apoptosis markers and an increased proportion of megakaryocytes with activated caspase-3 have been observed in bone marrow biopsies of ITP patients (28). TPO is the primary regulator of megakaryocytopoiesis and thrombopoiesis, supporting cell survival, cell cycling, and modulating apopotosis and cell cycle regulators (46). Patients with ITP, considering their often extremely low platelet count, characteristically exhibited lower than expected levels of endogenous TPO (eTPO) (56–58). It has also been observed that, although in the presence of similar platelet counts, eTPO levels in ITP patients were markedly lower than those observed in patients with aplastic anemia (5, 48, 59). The reason of this unexpected observation could be explained because of binding to Mpl on the increased MK mass with subsequent internalization and degradation or secondary to TPO bounds to platelets targeted for destruction (46). Furthermore, the observation of reduced TPO levels in the presence of low platelet count suggested impaired platelet production and failure to compensate for the thrombocytopenia (48). However, other studies comparing eTPO and platelets counts in ITP patients reported conflicting results (5, 6). Although the prevailing model of steady-state TPO regulation is based on the circulating TPO concentration inversely proportional “to Mpl mass,” a novel physiological feedback mechanism has been documented. Grozovsky et al., in 2015, demonstrated that the circulatory lifespan of platelets was determined by sialic acid loss that triggers platelet removal by the hepatic Ashwell–Morell receptor (AMR). The AMR is a transmembrane heteroligomeric glycoprotein complex composed of ASGPR1 (CLEC4H1, HL-1) and ASGPR2 (CLEC4H2, HL-2) subunits, which are highly conserved among mammalian species (7, 60). The AMR-mediated removal of desialylated aged platelets regulates TPO synthesis in the liver by recruiting janus kinase2 (JAK2) and transcrption3 (STAT3) phosphorylation (7). These data contribute to explain “the plasma TPO-level discrepancies” observed in human pathologic conditions, characterized by thrombocytopenia and liver disease, abovementioned. In fact, the antibody-mediated clearance of sialylated platelets would avoid the AMR platelet removal and, therefore, reduce TPO expression (7). These studies opened an interesting window of opportunity, indicating TPO receptor agonists (TPO-RA) as an alternative treatment option for children with chronic ITP. Clinical trials in children are ongoing and data are emerging on safety and efficacy of these agents (61). Although the autoantibodies-mediating accelerated platelet clearance from the circulation continues to be the central theme in the current understanding of ITP, its pathogenesis appears to be multifactorial, resulting as a complex interplay involving multiple components of the immune system (Figures 2 and 3).

#### The Role of Antigen-Presenting Cells

Following the exposition to the cell surface of “cryptic” antigens, the appearance of a wide spectrum of new peptides can therefore induce an “acquired cell recognition of new self-determinants” by the antigen-presenting cells (APC), playing a crucial role not only in the arising but also mainly in the progression of the ITP (62). These cells in normal conditions play the primary function of recognizing, binding to and internalizing protein antigens for endosomal proteolytic degradation. After these peptides complexed with MHC molecules are presented to T cells in conjunction with signaling costimulatory events (i.e., CD28/B7 and CD80/86 interactions between APC and T cell), and in the presence of appropriate cytokines, the T cell become properly activated (44). The local microenvironment and extrinsic stimuli can influence APC phenotype and function; in certain conditions, such as inflammation, APC abnormalities, in number and function, lead to abnormal processing or presentation of self antigens or inappropriate T cell activation. Dendritic cells, even referred as professional APC, are the most specialized among all leukocytes in the presentation of antigen. Peripheral blood DCs are classified in two broad groups according to their lineage, myeloid DCs (mDCs), often referred as conventional DCs, and plasmacytoid DCs (pDCs) (63). The mDC subset plays specific functions in the initiation of adaptive immune response, being critical for the activation of effector T cells and differentiation of naive CD4^+^ T cells into Th1 cells. The pDCs produce high levels of type I interferon (IFN-α/β/ω), reflecting their important function in antiviral immune responses (1, 63–65). The type I IFN is a pleiotropic cytokine not only with antiviral properties but also with capability to regulate pDC survival, mDC differentiation, mDC-mediated cytotoxic T cell responses, cross presentation, upregulation of costimulatory MHC molecules, and activation of natural killer (NK) and B cells (63, 66–69). Plasmacytoid DCs also display pro-inflammatory and immunosuppressive tolerogenic properties (70). Recently, low number of circulating pDCs has been shown in untreated patients with ITP (65). These authors explained this result as an abnormality of their distribution and suggested that pDCs could be mobilized into peripheral inflamed tissues, particularly to the spleen. Together with the pDC number alteration, also the dysfunction of DCs has been demonstrated to contribute to the development of ITP (71). In experiments performed by pulsing DCs from ITP patients and healthy donors, with autologous/allogeneic fresh and aged platelets, Catani et al. (71) showed that the DCs from patients stimulated T-cell proliferation in a greater degree than those one from healthy donors. They concluded that this might be due to an increased expression of the costimulatory antigen CD86 on the surface of DC patients (71). The importance of the ITP dysregulation of the APC-T cell cross talk has been reported by Zhong et al. (72).

#### The Role of Cytotoxic Cells

Cytotoxic T lymphocytes (CTLs) are cells able to kill neoplastic or infected cells with bacteria or viruses by programing these cells to undergo apoptosis (73). In ITP patients, with no identifiable anti-platelets autoantibodies, CTLs have been shown to increase both platelet and MK lysis, suggesting the role of these cells in mediating thrombocytopenia (31, 32, 74–76). Apoptosis and perforin/granzyme-mediated cytotoxicity constitute the main pathway used by CTLs to destruct autologous platelets (75). A variety of T cell and cytokine abnormalities have been observed in patients with ITP, suggesting that T cell-mediated peripheral platelet destruction and MK destruction/inhibition could be involved in the pathogenetic mechanism of thrombocytopenia (26, 27, 31, 44, 77–79). Interleukin-27 (IL-27), a member of IL-12 family, is a cytokine with multiple immunomodulatory functions, showing both pro-inflammatory and anti-inflammatory effects (80). Liu et al. demonstrated low plasma and mRNA expression levels of IL27 in active ITP patients, suggesting that this cytokine might be involved in the pathogenesis of ITP (81). Recent data suggested that IL-27 could inhibit platelet destruction by negatively regulating CTL cytotoxicity toward autologous platelets in ITP (80); this negative regulation was obtained by decreasing granzyme B expression whereas granzyme A and perforin were not affected (80). Based on these experiments, it has been suggested that IL-27 might have a therapeutic role in ITP patients (80). Thus, in addition to platelet-specific autoantibodies, also CTLs and aberrant cytokine profiles can be involved in platelet destruction, playing important role in the pathogenesis of ITP (73, 80).

#### T Follicular Helper Cells

T follicular helper cells are the specialized providers of B cell help; they are the key cell type required for the formation of germinal centers (GCs) and the generation of long-lived serological memory. TFHs have emerged to play a key role in regulating the humoral immune response that occurs with autoimmune diseases, infectious diseases, and tumors (82). TFHs are characterized by low expression levels of cytokines, IFN-γ, IL-4, and IL-17, characteristic of Th1, Th2, and Th17 cells respectively and by the expression of effector molecules that are critical for their development and function, including chemokine receptor 5 (CXCR5), inducible costimulator (ICOS), programed death-1 (PD-1), surface receptors of IL-21, IL-6, CD40, and transcription factors Bcl-6 and c-Maf (2, 83). It has been demonstrated that human splenic TFHs are the main producers of interleukin IL-21 (2), a cytokine playing a critical role in T cell and B cell homeostasis. In B cells, IL-21 signaling is involved in germinal center formation, Ig-secreting B cells, and antibody response (83, 84), while in CD4 T cells, it promotes the development of Th17 and TFHs themselves (83–85).

In ITP patients, splenic TFH frequency is higher than healthy donors and correlates with germinal center and plasma cell percentages that are also increased (2). Although the role of the inflammatory environment in the increase in TFH frequency could not be completely excluded, Audia et al. (2) strongly suggested that the expansion of TFHs could be involved in B-cell recruitment and differentiation in the spleen of ITP patients, through IL-21 secretion and by the interaction of CD154 with CD40, contributing to the development of plasma cells producing antiplatelet autoantibodies. Therefore, TFHs and associated molecules, such as IL-21 and CD40, could be new promising therapeutic targets for ITP patients.

### The Loss of Tolerance

#### The Th1/Th2 Balance

**Figure 4:**
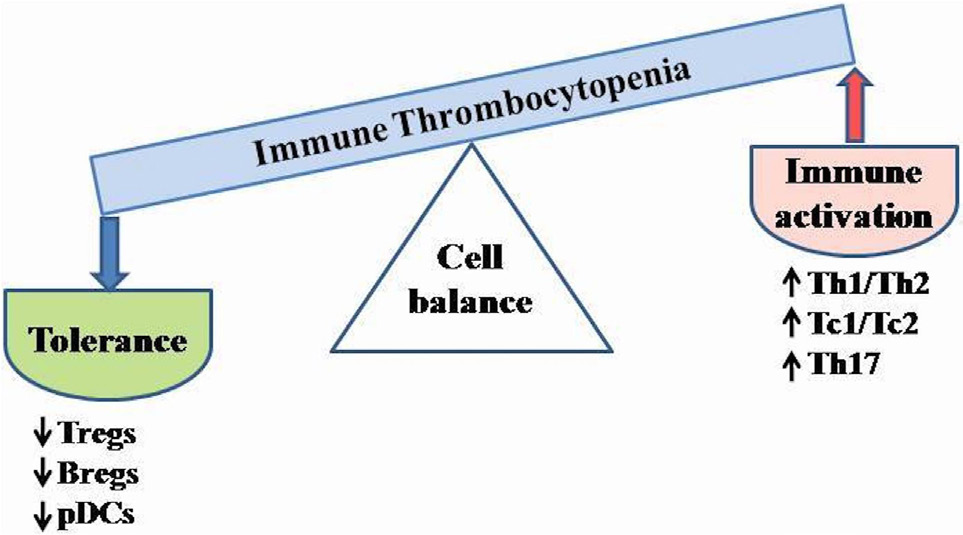
**Schematic cell imbalance in ITP**. Dysregulation of T cell activity and cytokine abnormalities are critical for ITP development. The unbalanced type 1/type 2 ratio leads to autoreactive B cell differentiation. Reduced number and/or impaired function of Tregs, Bregs, and tolerogenic DCs may contribute to enhance the immune activation. Bregs, B regulatory cells; pDCs, plasmacytoid dendritic cells; Tregs, T regulatory cells; Th, T helper cells; Tc, T cytotoxic cells.

Immune homeostasis is maintained *via* a balance of two types of responses, defined by cytokine secretion profiles. The responses of type 1, characterized primarily by IL2, IFNγ, TNFα, and TNFβ1 production, are involved in response to intracellular pathogens and generally promote pro-inflammatory, cell-mediated, complement fixing phenotypes. On the other hand, type 2 reactions, characterized by IL4, IL5, IL6, IL10, and IL13 cytokine production, function in the fight against extracellular pathogens, and typically elicit an immediate-type hypersensitivity response (43). The polarization of the immune system toward either type 1 or type 2 immunity is dependent on the level of the cytokines. In ITP, type 1/type 2 ratio is unbalanced, shifted toward a type 1 phenotype, favoring autoreactive B cell development (a Th2 phenotype being observed in patients during remission or during intravenous immunoglobulin treatment) (1, 43, 86, 87). This has been attributed to the reduction of peripheral Th2 cells, of Tc2 cells and of CD25^bright^Foxp3^+^ Tregs either in number or in function (30, 43, 87–90). Zhong et al. (72) showed that the CD16^+^ monocyte subset from the peripheral blood of chronic ITP promoted the expansion of the Th1 subset *via* IL12 secretion, while inhibited the proliferation of Th17 cells and the induction of Tregs. The Th1/Th2 balance in ITP is showed in Figure 4.

#### Treg and Th17 Compartments

Tregs are a subpopulation of T cells specialized in immune suppression and contribute to the maintenance of peripheral immune tolerance; their defects are thought to play a role in the pathogenesis of various autoimmune diseases (91). In the last decade, data from numerous studies have demonstrated a dysregulation of Treg cell population in ITP (29, 30, 91–102). Treg deficiency was reported in both adults and children with ITP (91): the lowest Treg frequency was detected in patients with acute phase of the disease and/or with low platelet count, while Tregs increase was observed in remission phase. Interestingly, a failure of the cross communication between DCs and Tregs in patients with ITP has been reported (103). These authors showed that the decreased expression and/or activity of the immunomodulatory enzyme indoelamine 2,3-dioxygenase 1 (IDO1) by DCs, contributed to the reduced conversion from T cells into Tregs, suggesting the importance of the cross talk between DCs and the adaptative immune system in the pathophysiology of immune thrombocytopenia (103). T cell-related pro-inflammatory cytokines may play a pivotal role in immune dysregulation during active ITP. In the past years, increased levels of macrophage colony-stimulating factor (M-CSF) (causing a stimulus for to the platelet antigen presentation by macrophages to T cells), reduced levels of transforming growth factor (TGF)-β production have been described in children with chronic ITP (104). IL10 is a cytokine with anti-inflammatory properties; initially described as a product of Th2 cells able to inhibit cytokine synthesis of Th1 cells (105), IL-10 is now known to be produced by many different types of cells, including Tregs (106). Based on data showing that IL10 producing Tregs contributed to the effective control of several autoimmune diseases (107), a recent study investigated the role of this cytokine in newly diagnosed ITP patients (106). The Authors identified a numerical and functional defect of Tregs associated with an excessive activation T effector cells, suggesting that the assessed insufficient secretion of IL-10 could compromise the inhibitory capability of Tregs against T effectors cells and play a major role in the exuberant CD4^+^ T cell immune response of ITP (106). More attention has been deserved to IL17, a cytokine produced by Th17 cells, a newly defined CD4^+^ Th subset; Th17 cells modulate the pro-inflammatory response by producing, as well as IL17, also IL6, IL21, tumor necrosis factor (TNF), and other mediators (108–110). Thus, while Treg cells play a fundamental role in the maintenance of immune tolerance to prevent autoimmune disease, Th17 cells play the opposite role. Indeed, it has been demonstrated that Th17 cells are more potent than Th1 cells in inducing autoimmune disorders (111–113). Several reports support a role for IL17 in the pathogenesis of ITP. Rocha et al. (110) suggested that increased levels of IL17 and of Th17-related cytokines found in adult patients with chronic ITP might contribute to the disease pathogenesis. The Treg/Th17 imbalance has been found associated with disease activity among adult ITP patients, implying an association between Th17 cell elevation and the development of ITP and suggesting a prognostic role of this cytokine (114). Wang et al. (115) found that IL17 levels were elevated in pediatric patients with chronic ITP and positively correlated with INFγ expression, suggesting that both Th17 and Th1 might be simultaneously involved in the dysregulation of cellular immunity observed in pediatric patients. However, other groups did not report significant differences either in the serum levels or in the expression of IL17 on peripheral blood mononuclear cells between ITP patients and normal controls (116, 117), arguing that IL17 might not play an important role in the pathogenesis of adult patients with chronic ITP. These conflicting results concerning the IL17 role underline the variability in the clinical phenotype and evolution of the disease duration among the ITP population. The IL23/IL17 axis is critical for the development of inflammatory diseases. IL23, which is mainly secreted by APCs, is the pivotal mediator responsible for the differentiation of naive CD4+ T cells into Th17 cells; the importance of the IL23 cytokine and of its receptor in both Th17 maturation and function has been demonstrated (113, 118, 119). Several studies reported that the IL23/Th17 pathway was strongly involved in the pathogenesis of many autoimmune diseases, such as rheumatoid arthritis (120), multiple sclerosis (121), primary biliary cirrhosis (122), and inflammatory bowel disease (123). However, less is known with regard to the levels of expression and synthesis of these two cytokines in patients with ITP. The role of IL23/Th17 pathway in adult ITP patients has been recently investigated (113). These Authors, found higher levels of IL17 and IL23 in patients with ITP than in controls and a decrease of both cytokines after effective treatment, suggesting that IL23 was engaged in the development of ITP through enhancement of the Th17 response.

#### The Role of Bregs

Interestingly, Bregs are a distinct B cell population with potent immunoregulatory properties; among them, the most well subset characterized is the IL10-producing subset (B10 cells) (1, 124, 125), which plays an important role in autoimmune diseases (1, 87, 126, 127). Recent findings showed that IL10 produced by B10 cells is required in combination with different costimulatory molecules for the differentiation and maintenance of Tregs and for inhibition of Th17 cells (87, 128, 129). Recently, it has been demonstrated that a dysregulation of Breg compartment was an additional defect in the immune network in ITP patients (1, 87). Hua et al. (87) demonstrated that the number of B10 cells positively correlated with both Treg count and Treg/Th17 ratio, suggesting that the ability of these cells to regulate functional T cell subsets might be impaired in ITP patients.

### Genetic Susceptibility

Only a small number of individuals develop ITP after exposure to infectious agents. Such variability could be explained by differences in genetic factors associated with the host defenses to pathogens, such as genes encoding inflammatory cytokines (9). In the recent past, genomic studies have allowed the knowledge of genetic factors influencing the development of ITP chronic pattern. Abnormal polymorphisms have been identified within the genes for several inflammatory cytokines. Satoh et al. (8) identified polymorphisms at TNFβ (+252 GG) gene. As TNFβ (+252) influences the capacity of T and B cells to produce TNFβ, by checking the autoreactive T- and B-cell responses to platelet membrane antigens, the Authors suggested that these polymorphisms might play a role in an individual’s susceptibility to ITP, by promoting the specific autoantibody response. Interestingly, a recent study demonstrated that the heterozygous variant (AG) genotype of TNFβ was associated with persistent ITP (130). Reports about TNF-α-308 gene polymorphisms are conflicting in both adult and pediatric ITP (130–132). IL4 (VNTR intron 3) and IL10 (−627) polymorphisms have also been associated with the pathogenesis of ITP and believed to contribute to the susceptibility of developing ITP (133–135). CNR2 is a gene encoding CB2, a cannabinoid receptor expressed on various immune cells and involved in immune regulation by modulating Th1 and Th2 balance and cell migration and by inhibiting pro-inflammatory cytokine production (9, 136, 137). CNR2 functional variant (Q63R) has been described to influence childhood ITP toward the chronic course of the disease (9, 138). Recent works demonstrated that stress-related pathways might contribute to the pathogenesis of ITP. In particular, over expression of vanin-1 (VNN-1), an oxidative stress sensor, has been demonstrated to be strongly associated with progression to chronic disease in both pediatric and adult patients (9, 139–141).

## Conclusion

Since the beginning of the century, new insights have been provided about the pathogenesis of ITP. Consequently, the nomenclature concerning both its definition and evolutionary steps are modified. Although the accelerated platelet destruction by platelet autoantibodies is considered the hallmark of ITP pathogenesis, research advances highlight the complex immune mechanism underlying the disease. The continuous fine understanding of the contribution of each immune defect (e.g., the checkpoint tolerance defect variously involved in ITP patients) could explain the clinical diversity and the different tendency to perpetuate the disease observed in the ITP population and similarly open new windows of opportunities for targeted therapy and “tailored treatment.”

## Author Contributions

All Authors contributed to the work presented in this paper. RC wrote the paper. All Authors reviewed the paper, and provided approval of the final version.

## Conflict of Interest Statement

The authors declare that this work was conducted in the absence of any commercial or financial relationships that could be construed as a potential conflict of interest.
